# Gut Microbiota beyond Bacteria—Mycobiome, Virome, Archaeome, and Eukaryotic Parasites in IBD

**DOI:** 10.3390/ijms21082668

**Published:** 2020-04-11

**Authors:** Mario Matijašić, Tomislav Meštrović, Hana Čipčić Paljetak, Mihaela Perić, Anja Barešić, Donatella Verbanac

**Affiliations:** 1 Center for Translational and Clinical Research, University of Zagreb School of Medicine, 10000 Zagreb, Croatia; hana.paljetak@mef.hr (H.Č.P.); mihaela.peric@mef.hr (M.P.); 2 University Centre Varaždin, University North, 42000 Varaždin, Croatia; tomislav.mestrovic@gmail.com; 3 Division of Electronics, Ruđer Bošković Institute, 10000 Zagreb, Croatia; anja.baresic@irb.hr; 4 Faculty of Pharmacy and Biochemistry, University of Zagreb, 10000 Zagreb, Croatia; dverbanac@pharma.hr

**Keywords:** gut microbiota, inflammatory bowel disease (IBD), mycobiome, virome, archaeome, eukaryotic parasites

## Abstract

The human microbiota is a diverse microbial ecosystem associated with many beneficial physiological functions as well as numerous disease etiologies. Dominated by bacteria, the microbiota also includes commensal populations of fungi, viruses, archaea, and protists. Unlike bacterial microbiota, which was extensively studied in the past two decades, these non-bacterial microorganisms, their functional roles, and their interaction with one another or with host immune system have not been as widely explored. This review covers the recent findings on the non-bacterial communities of the human gastrointestinal microbiota and their involvement in health and disease, with particular focus on the pathophysiology of inflammatory bowel disease.

## 1. Introduction

Trillions of microbes colonize the human body, forming the microbial community collectively referred to as the human microbiota. Spanning all three domains of life, bacteria, eukaryotes, and archaea, as well as viruses, the human microbiota plays an important part in human physiology and health maintenance by helping in energy harvest, supporting the immune development, and providing protective, structural, and metabolic functions essential for the human body.

Recent advancements in next-generation sequencing and computational technologies have provided opportunities to investigate the structure and function of microbial communities associated with various body sites. As bacteria are the most abundant component of the microbiota, the vast majority of studies over the last decades focused primarily on the composition of bacterial microbiota and its effects on human health and disease. On the other hand, research on human fungal (mycobiome), viral (virome), and archaeal microbiota (archaeome) is still in its infancy. Nevertheless, recent studies revealed that these non-bacterial microbial populations are also dynamic communities, interacting with one another and playing a vital role in host wellbeing [1,2,3,4].

**Figure 1:**
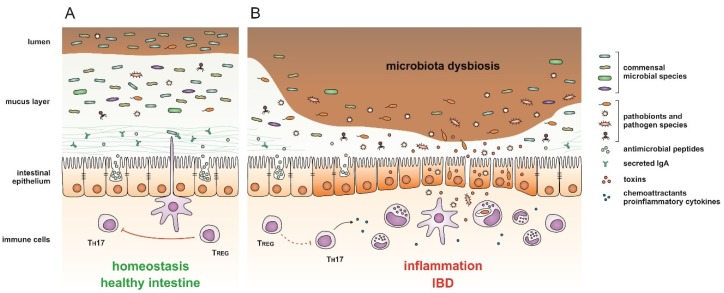
A schematic representation of intestinal mucosa in (**A**) healthy and (**B**) inflammatory bowel disease (IBD)-affected individual. A thick mucus layer covers the epithelium of the healthy intestine. Microbiota is dispersed throughout the outer mucus layer, while the inner layer is thick and resistant to penetration due to antimicrobial peptides secreted by epithelial cells, and immunoglobulin A (IgA). Commensal microbiota suppresses the proliferation of pathobionts and pathogens, tuning the host responses towards immunological tolerance and intestinal homeostasis. Various factors can disrupt the composition of intestinal microbiota, resulting in dysbiosis and excessive reproduction of pathogenic microorganisms. These species produce and secrete toxins, thinning the protective mucus layer, damaging the intestinal mucosa, and increasing intestinal permeability. The microbes can gain access to epithelial cells and mucosal tissue inducing the imbalance of the T_REG_/T_H_17 axis and thus a strong inflammatory response by the host immune system leading to or aggravating IBD.

The gastrointestinal tract is the most densely populated microbial niche of the human body. Despite its well-established host-beneficial functions, intestinal microbiota has been implicated in various pathological conditions, including inflammatory bowel disease (IBD) and its two main entities: Crohn’s disease (CD) and ulcerative colitis (UC). Numerous studies demonstrated that chronic inflammation of the intestinal mucosa in IBD is associated with alterations in microbial community structure and function, termed “dysbiosis” (Figure 1). The dysbiotic bacterial microbiota has been extensively characterized in IBD [5,6], while the role of other microbiota constituents, as well as their interactions within the intestinal microbiota, remain unclear.

## 2. Human Mycobiome

Fungi are ubiquitous in the environment and a part of all Earth’s ecosystems [7]. In addition, a diverse population of commensal fungi has been recognized as a fundamental component of the human body, co-existing with other microbes within the human microbiota [8]. In contrast to the vast number of studies on the bacterial communities of the microbiota conducted in the last decades, the fungal constituents of the microbiota, the mycobiome, received much less attention. Still, recent research acknowledged human mycobiome as a dynamic community, responsive to environmental and pathophysiological changes, and playing a vital role in host metabolism, as well as maintenance of host immune homeostasis [3,8,9,10].

Early research of human mycobiome was based on culture-dependent techniques for the identification and characterization of commensal fungal communities. While the new molecular culture-independent next-generation sequencing (NGS) techniques proved very effective for analyzing the bacterial component of microbiota, the DNA-based sequencing studies of the human mycobiome are faced with several limitations. Fungi account for a relatively small percentage of the human microbiota, with 10^5^ to 10^6^ fungal cells per gram of fecal matter (compared to 10^11^ bacterial cells per gram) [11] and only 0.1% of the 9.9 million reference genes in a current human gut microbial metagenomic reference catalog are reported to be of eukaryotic origin [12,13]. Additionally, the identification of composition and diversity of the fungal community is influenced by the nucleic acid isolation method [14], the choice of sequencing primer pairs [15], as well as different sequencing technologies [16,17] and bioinformatics pipelines [18,19]. Finally, the incomplete databases for taxonomic assignment and annotation of fungal genomes present a serious difficulty in studying the human mycobiome [15]. The usual molecular target for identifying fungi are the internal transcribed spacer (ITS) regions of ribosomal RNA genes. As the ITS regions are highly divergent among fungi, these regions are often sufficiently different to classify fungi to the species level. In 2012, ITS was designated as the universal DNA barcode marker for the kingdom Fungi [20], although this approach revealed potential PCR biases [21,22]. A recent study proposed adding translational elongating factor 1α (TEF1α) as a secondary barcode to the ITS barcode in order to increase the taxonomic resolution power and enhance the accuracy of fungal species identification [23]. On the other hand, the study comparing 18S rRNA screening to ITS sequencing showed higher sensitivity of 18S rRNA RT-PCR combined with SANGER sequencing, as this method detected fungal communities in several samples which were ITS negative [24]. Currently, there is no consensus on the best methodological approach for identifying human mycobiome, and consequently the results of studies using different methods vary.

Human mycobiome inhabits the gastrointestinal tract but also skin, respiratory tract, genitourinary tract, as well as other mucosal surfaces in the host. The gastrointestinal tract is the most studied fungal niche in humans. Reports suggest that the human gut is populated by three fungal phyla, *Ascomycota*, *Basidiomycota*, and *Zygomycota* [25,26], with the “core” 10 genera identified in the majority of gastrointestinal tract samples consisting of *Candida* (particularly *C. albicans*), *Saccharomyces* (particularly *S. cerevisiae*), *Penicillium*, *Aspergillus*, *Cryptococcus*, *Malassezia* (particularly *M. restricta*), *Cladosporium*, *Galactomyces*, *Debaryomyces*, and *Trichosporon* [25,27]. The composition of gut mycobiome seems to be dynamic over time and far more variable than the composition of bacteria, both in humans [28] and in mice [29]. Most studies consider fungi as commensal organisms in the gut, acquired early in life [30]. This has recently been challenged claiming that fungi do not routinely colonize the gastrointestinal tract of healthy adults [31], instead postulating that all fungi identified in the human stool samples could be explained by their presence in the mouth or the diet. Indeed, diet is perceived as a crucial factor affecting the composition and variability of gut mycobiome [32]. For instance, gut mycobiome content was found to considerably differ between individuals having different dietary patterns, i.e., vegetarians and people on a conventional Western diet [25,32]. Additionally, reports suggest that the abundance of *Candida* in the gut positively correlated with high carbohydrate diets, and inversely correlated to consumption of total saturated fatty acids, while recent intake of short-chain fatty acids reduced the abundance of *Aspergillus* [26]. Another notable finding of this study was the co-occurrence of *Candida* with particular bacterial (*Prevotella* and *Rumminococcus*) and archaeal genera (*Methanobrevibacter*), providing support for the interkingdom syntrophic relationships in host metabolism.

One of the first indications that fungi play a role in modulating gut homeostasis is the use of *Saccharomyces boulardii* as a constituent of herbal medicine traditionally utilized in Southeast Asia to reduce the severe diarrhea in patients with cholera. *S. boulardii* is still prescribed as a probiotic to prevent diarrhea and intestinal colonization with *Clostridium difficile* following antibiotic therapy [33,34] and is efficient in preventing recurrent *C. difficile* infections [35]. The positive effects of *S. boulardii* come from inactivating pathogen toxins and directly inhibiting the growth and invasion of intestinal pathogens [36,37], as well as boosting the host immunity and exerting anti-inflammatory functions in ulcerative colitis [38,39], Crohn’s disease [38,40], and *C. difficile* colitis [41]. A recent report suggests beneficial effects of another probiotic yeast, *Candida kefyr*, in reducing the severity of colitis in animal models by decreasing the abundance of *Bacteroides* and lowering IL-6 production, thus attenuating inflammation in the intestine [42].

Although fungi can exert beneficial effects to host health, the disturbance of gut mycobiota was also implicated in various gastrointestinal diseases. A recent study demonstrated no significant changes in mycobiome richness between obese and non-obese subjects; however, some specific compositional differences were noted. The most prevalent genus in non-obese individuals was *Mucor*, with its abundance significantly higher in non-obese individuals, and inversely correlated with metabolic markers of obesity [43]. In colorectal cancer (CRC), an alteration of fungal composition and ecology was observed, characterized by an increased *Basidiomycota*/*Ascomycota* ratio, depletion of *S. cerevisiae*, as well as enrichment of *Rhodotorula*, *Malassezia*, and *Acremonium* genera along with several *Aspergillus* species (including *A. flavus*, a major producer of highly toxic carcinogen aflatoxin), suggesting their possible contribution towards CRC pathogenesis [44]. Insights into gut mycobiota playing a role in IBS were also reported. Decreased fungal diversity and dysbiosis were found in IBS patients, correlating mycobiota signature with visceral hypersensitivity, which is considered as one of the major pathophysiological features of IBS [45]. Interestingly, treatment with fungicides could recover the visceral hypersensitivity to normal levels [45]. This finding is in accordance with a previous study that reported yeast-free diets and antifungal treatments as helpful for IBS subjects [46]. In addition, *S. boulardii* was found to be effective in improving symptoms and the quality of life in IBS patients [47].

The majority of research on the effects of gut mycobiota in gastrointestinal diseases was however concentrated on intestinal inflammation and IBD. Even before the advent of molecular methods and NGS, increased levels of anti-*S. cerevisiae* antibodies (ASCA) were commonly found in the serum of CD patients, suggesting the host’s immune responses toward intestinal fungi [48]. These antibodies, raised against mannan, a component in the fungal cell wall, were soon identified as a reliable diagnostic biomarker for CD and predictors of the disease course [49,50]. ASCA also recognize many other fungi, including *Candida* [51]. Indeed, reduced fungal diversity and significantly increased abundance of specific *Candida* species were found in pediatric IBD patients [52]. Sokol et al. report a similar finding in adult subjects with IBD: a decrease in gut mycobiome biodiversity and elevated *Basidiomycota*/*Ascomycota* ratio, mainly due to the increased prevalence and abundance of *C. albicans* and reduction of *S. cerevisiae* [53]. Additional studies confirmed an increased representation of *Candida* species in IBD, namely *C. tropicalis* in familial CD [54], as well as *C. glabrata* in colonic biopsy samples from patients with CD [55]. Besides elevated *Basidiomycota*/*Ascomycota* ratio in IBD patients in comparison to healthy controls and in IBD flares vs. IBD remission [53], fungal dysbiosis in IBD patients is also characterized by increased levels of *Gibberella moniliformis*, *Alternaria brassicola*, *Aspergillus clavatus*, and *Cystofilobasidiaceae* [55], while *Saccharomyces cerevisiae* and *Malassezia sympodialis* are markedly decreased [53]. Additionally, studies confirm fungal burden is increased in both CD and UC [55,56], with the fungal cells translocating trough the intestinal barrier during the chronic stage of colitis [56,57].

Some of the studies simultaneously analyzed both the fungal and bacterial microbiota revealing that the intestinal microbial network was different in IBD patients when compared to healthy individuals. Sokol et al. identified positive correlations between the decreased abundance of *S. cerevisiae* and reduction of several bacterial genera, such as *Bifidobacterium*, *Blautia*, *Roseburia*, and *Ruminococcus*. The total number and the intensity of fungal–bacterial associations were increased in UC, with distinct interactions potentially involved in the inflammatory processes. On the other hand, weaker fungal–bacteria correlations were found in CD when compared to healthy volunteers, implying disrupted connections between two kingdoms in this disease [53]. A study by Hoarau et al. reported elevated levels of *C. tropicalis* positively correlated with *Serratia marcescens* and *Escherichia coli* in CD. Moreover, in vitro experiments confirmed these species form thicker mixed biofilm than any of the species generates individually, creating a commensal niche additionally enriched in fungal hyphae, a form of growth usually implicated in pathogenic conditions [54]. The fact that interactions between gut bacteria and fungi are closely associated with disease was also investigated in mouse models of dextran sulfate sodium (DSS) induced colitis. Qiu et al. found that inflamed mouse intestine contained increased fungal burden in the mucosa, but decreased in the feces. The dysbiosis was characterized by elevated *Wickerhamomyces*, *Alternaria*, and *Candida*, together with reduced *Cryptococcus*, *Phialemonium*, and *Wallemia*, and unidentified *Saccharomycetales* genus [57]. The study further shows mice with fungi depleted by fluconazole treatment exhibited aggravated colitis, in contrast to bacteria-depleted mice, that showed alleviated intestinal inflammation and a trend of disease remission. This finding suggests that bacteria are the major driving force in acute inflammation and fungi may act as a counterbalance in maintaining the microbial homeostasis in acute colitis. In chronic recurrent colitis however, fungi may aggravate the disease severity and translocate into locations outside the gut [57]. A recent study by Sovran et al. identified opposing effects of administrating *C. albicans* or *S. boulardii* to mice with DSS-induced colitis, resulting in increased disease severity or reduced disease symptoms, respectively. However, broad-spectrum antibiotic treatment protected the mice from colitis and *C. albicans* had no pro-inflammatory effect when administered to mice with disrupted bacterial microbiota, suggesting bacteria are essential for the development of colitis and *C. albicans* requires the presence of specific bacteria that trigger the intestinal inflammation to increase the disease intensity. On the other hand, mice with depleted *Enterobacteriaceae* exhibited normal susceptibility to colitis, but neither *C. albicans* nor *S. boulardii* could exert disease-modulating effects in this experimental setting. After reintroducing *Enterobacteriaceae*, both *C. albicans* and *S. boulardii* recovered their effects in severity of colitis [58].

The host immune system recognizes fungi using pattern recognition receptors (PRRs), with the resulting host responses ranging from tolerance to inflammation. The key PRR for coordinating host response to fungi is Dectin-1 (CLEC7A), a C-type lectin receptor that recognizes β-glucans in the fungal cell wall [59]. Dectin-1 activates macrophages and dendritic cells, initiates phagocytosis of fungi, and induces signaling cascade via caspase-associated recruitment domain-containing protein 9 (CARD9) and NF-kB to produce pro-inflammatory cytokines. A recent study demonstrated a central role of Dectin-1 in regulating the severity of inflammation in mouse models of DSS-induced colitis [60]. Dectin-1 deficient mice were found to develop more severe colitis, due to the overgrowth of opportunistic fungi (i.e., *Candida* and *Trichosporon*), while treatment with antifungal drug fluconazole ameliorated the disease [60,61]. The same study revealed that a polymorphism in the Dectin-1 gene was associated with increased severity of disease in patients with UC [60]. Recent research also identified CARD9 as the key downstream signaling molecule for the induction of immune response to fungi [62]. CARD9-deficient patients are especially susceptible to fungal infections and polymorphism in CARD9 gene is associated with a higher risk of developing IBD [63]. Interestingly, *Candida* overgrowth, which is one of the characteristic features in IBD patients, could not be positively correlated with CARD9 polymorphism [64]. Instead, *Candida* was hardly detectible in CARD9 deficient mice, suggesting this taxon was not the driver of dysbiosis as in dectin-1 deficient animals [65]. IL-17 and IL-22 were also found to affect commensal fungal communities. A clinical study revealed secukinumab, an IL-17A antagonist, was associated with exacerbations in patients with CD, identifying the higher rate of fungal infections in treated subjects [66]. Both IL-17 and IL-22 might act as inducers of antimicrobial peptides (AMPs) in epithelial cells and were reported as protective against mucosal fungal infections [67,68].

## 3. Human Virome

Viruses are the most abundant and widespread biological entities on Earth [69] and integral members of the human microbiota. The entire assembly of eukaryotic and bacterial viruses (bacteriophages) populating the human body was termed the human virome. The virome includes double-stranded DNA (dsDNA), single-stranded DNA (ssDNA), as well as RNA viruses. Significant research effort in recent years was devoted to characterize this diverse community and appraise its impact on the host wellbeing [70,71,72]. Viruses are found in numerous human body sites, with the intestine being the most extensively populated and studied niche. Intestinal virome is large and diverse, but nonetheless stable and highly personalized [1,73], established in the earliest period of human life simultaneously with the colonization of bacteria and other constituents of the gut microbiota [74]. The gut virome in the neonatal period predominantly consists of bacteriophages, with only a small fraction of eukaryotic viruses, and akin to the bacterial microbiome, it significantly varies across individuals [75]. In contrast to research on bacteria and fungi, no universal marker gene can be utilized to study viruses and the majority of obtained sequences do not exist in publicly available databases (“viral dark matter” [76,77]), so the usual methodological approach of virome research is metagenomics.

The healthy human intestine is a home to approximately 10^15^ bacteriophages, outnumbering the commensal bacteria by a factor of 10 [71,78], with the “core phageome” predominantly consisting of dsDNA viruses from the *Caudovirales* order (*Myoviridae*, *Podoviridae*, and *Siphoviridae* families) and ssDNA viruses of the *Microviridae* family [79]. A recent study identified a novel type of phage, crAssphage, as the most prevalent human-associated virus, accounting for up to 90% of the reads from human fecal viral metagenomes and about 22% of the reads in the total metagenome [80]. CrAssphage and novel crAass-like phages are associated with *Bacteroidetes* bacterial phylum and are likely to become a family within the *Caudovirales* order [81,82]. The phages usually exhibit temperate lifestyle and integration into bacterial hosts as prophages, however, environmental stressors can induce the lytic cycle resulting in viral replication and destruction of host cells. Thus, the intestinal virome is closely linked to the prokaryotic microbial communities not just by sharing a common niche, but also by contributing to the ecosystem dynamics as well as by providing various genetic elements such as virulence factors or antibiotic resistance genes while integrated as a prophage [83]. Accordingly, there is mounting evidence that the community of phages in the gut is altered when dysbiosis-related disorders are considered, such as IBD and colorectal cancer. In addition, a shift from lysogenic to lytic replication in the population of temperate phages may be linked to the development of IBD [84].

The exact role of human gut virome in the etiology of IBD has yet to be elucidated, as not only our understanding of the virome is still fragmented and without clear links to different inflammatory conditions, but there is also an inherent complexity of IBD as a prototypic multifactorial disease [1,85]. Still, recent research provided some rationale for considering virome as a potentially important stakeholder in the development of chronic inflammatory conditions of the gut [86]. One of the first studies that linked gut virome dysbiosis with IBD pathogenesis clearly showed the increased abundance of phages infecting bacterial orders *Alteromonadales* and *Clostridiales*, including bacterial species *Clostridium acetobutylicum*, as well as elevated numbers of *Retroviridae* family representatives, in subjects with CD [87]. In this paper, authors also pinpointed viral biomarkers associated with the disease and hinted the importance of interactions between viral and bacterial communities in the gut [87]. A large study by Norman et al. observed disease-specific changes and reduced diversity of the gut virome in both CD and UC, revealing the primary difference in the IBD-associated virome was the increased richness of *Caudovirales* phages on a taxonomic level; however, the exact viruses that may give rise to such change were different in CD when compared to UC [88]. A dataset used for this study was recently reanalyzed, confirming IBD-specific changes in the virome, loss of the “core phageome” and increased abundance of induced temperate phages in patients with CD [84]. However, in contrast to the previous report, no changes in viral richness and overall viral alpha diversity were demonstrated. Furthermore, the changes in the composition of virome mirrored shifts in bacterial composition, while both virome and bacteriome alterations were more pronounced in patients with CD when compared to UC, reflecting the disease severity. Hence, integrating both bacteriome and virome assessment offers higher classification power between healthy and diseased states in IBD [84]. In addition, Fernandes et al. revealed how minor patterns and differences in gut virome may be used to differentiate pediatric patients with IBD from healthy controls. In this study, most pronounced changes have been observed in the relative abundance of *Caudovirales* order of phages, with a substantially lower number of strains in the *Microviridae* family in patients with CD compared to healthy controls [89]. Similarly, the expansion of the *Caudovirales* was observed in another study, together with decrease in richness and diversity in patients with UC [90].

Recent animal studies corroborated the role of enteric phages in IBD. By using murine models, Seth et al. showed that increased viral richness and increased alpha diversity exhibit a positive correlation with gut dysbiosis and the level of proinflammatory cytokines in the serum [91]. Similarly, another study showed the increasing bacteriophage levels (primarily *Escherichia*, *Lactobacillus*, and *Bacteriodes*-infecting phages) in germ-free mice may alter the state of mucosal immunity and exacerbate colitis by inducing the production of IFN-γ via TLR9 [92]. Additionally, recent research suggests that *Spounaviridae* phage subfamily, as well as phages that target *Streptococcus* and *Alistipes* may serve as informative disease markers for murine colitis analogous to IBD [93]. Interestingly, this study did not reveal increased levels of bacterial hosts, suggesting the abundance of phages is not always correlated with host abundance and that inflammation might be caused by induction of phage replication and excision. However, the most critical finding was the overlap of intestinal phage metagenomes in the mouse model and human studies, indicating that the mouse colitis model could be suitable for studying human disease [93].

Bacteriophages were recently proposed as a therapeutic option for treating CD [94]. A cocktail of phages was reported to reduce symptoms in the mouse model of dextran sulfate sodium (DSS)-induced colitis, as well as significantly reduce fecal adhesive-invasive *E. coli* (AIEC). AIEC was shown to colonize the ileal mucosa of CD and correlate with disease location, activity, and postoperative recurrence [95]. In fact, the phage cocktail was found to target AIEC in homogenates of ileal biopsies taken from CD patients [94], and the preparation is currently under evaluation in a clinical trial.

Although most research concentrated on phage virome, eukaryotic virome is also proving to have a substantial role in human health and disease; therefore, both should be considered as distinct, but related entities. The human intestinal mucosa is increasingly colonized with eukaryotic viruses from birth to 2 years of age, suggesting that the eukaryotic virome is established through environmental exposure [74]. The human eukaryotic virome consists of *Adenoviridae*, *Anelloviridae*, *Astroviridae*, *Parvoviridae*, *Picornaviridae*, and *Picobirnaviridae* families, demonstrating that viruses, which are usually considered as pathogens or opportunistic pathogens, frequently populate the human intestine [74]. These viruses can exert symptomatic manifestations or can remain latent for a long time, also providing beneficial effects for the host [86,96].

A growing body of data confirms that perturbations in eukaryotic virome are linked to the pathogenesis of IBD [86]. Zuo et al. employed deep sequencing techniques to discern alterations of the gut virome in patients with UC, showing an increased abundance of *Pneumoviridae* in UC patients compared to the control group, while the opposite was found for *Anelloviridae* family [90]. A study that analyzed colon samples of IBD patients and healthy controls showed that IBD was characterized by increased levels of *Herpesviridae* family, as well as augmented expression of pertaining endogenous viral sequences [97]. Larger studies are warranted to further elucidate this relationship, although the role of certain herpesviruses in IBD development and disease exacerbations was already described [98]. Recently, Ungaro et al. utilized a thorough metagenomic analysis of gut mucosa on a large cohort of treatment-naïve IBD patients to explore potential viral signatures and triggers [99]. They found a high abundance of *Hepadnaviridae* family (together with the protein HBx) in patients with UC, and an increased abundance of *Hepeviridae* family when compared to controls. On the other hand, diet-related *Polydnaviridae* and *Tymoviridae* viral families were less enriched in patients with UC, and the same was valid for *Virgaviridae* in patients with CD [99]. The role of eukaryotic viruses as potential triggers of gut inflammation has been studied in animal models as well, mostly concentrating on *Norovirus* as a contributor to intestinal inflammation [100,101].

Since it is clear that many viral families are potential players in the development of inflammatory processes in the gut, further studies are needed to precisely identify possible viral triggers, principally by appraising metagenome signatures of those with the disease. Nonetheless, there are many challenges in studying human virome, and the biggest may be the sample composition (as most habitually a fecal specimen is used) [102]. Extensive chemical, enzymatic, and mechanical processing is indispensable for eliminating cellular DNA and dietary elements to enrich the virome fraction [103]. This issue is even more pronounced when studies of mucosal virome are pursued, and where DNA yields are substantially lower [104]. Therefore, akin to other metagenomic approaches, it is pivotal to balance sequencing depth, sequencing chemistry, and read length. However, these challenges are bound to be solved, and as gut virome research becomes standardized and expands even further, opportunities will arise to explore virome-directed treatment approaches for IBD, clinical testing of aforementioned phage preparation being the first step in that direction.

## 4. Human Archaeome

Archaea are a separate domain of life, distinct from Bacteria and Eukarya [105]. Despite being morphologically similar to bacteria regarding their shape, size, and unicellular organization, archaea feature many characteristics that are more closely related to those of eukaryotes, such as DNA replication and repair mechanisms, RNA transcription, and protein translation machinery [106]. Although sharing properties with both Bacteria and Eukarya, archaea exhibit unique characteristics not present in either of these two domains. The archaeal cell walls do not contain peptidoglycans, but are composed diverse structures including methanochondroitin and pseudomurein [107], while their cell membranes are composed of L-glycerol-ether/isoprenoid lipids (in contrast to D-glycerol-ester/fatty acid lipids in bacterial and eukaryotic membranes), enhancing membrane stability and rigidity [108]. Finally, archaea use distinctive metabolic pathways that can utilize sunlight, organic, and inorganic substrates as energy sources [109]. Due to their biochemical and metabolic advantages, archaea are capable of populating a broad variety of habitats. The first archaea identified were extremophile species thriving in severe environments; however, with the advanced detection tools and molecular methods, archaea were also found in moderate climates, constituting a considerable portion of the Earth’s microbial biomass [109]. Furthermore, archaea were detected in plants [110] and in animal digestive tracts as part of the commensal microbiota [111,112].

Miller et al. discovered and characterized the first archaeon in human feces, *Methanobrevibacter smithii*, over 35 years ago [113]. As the research on the human microbiota focused almost exclusively on the commensal bacteria, the insight into the human archaeome during the following decades was limited to only three archaeons identified in human stool samples: *M. smithii*, [113], *Methanosphaera stadtmanae* [114], and *Methanomassiliicoccus luminyensis* [115], and one from oral mucosa: *Methanobrevibacter oralis* [116]. Recently, the application of molecular tools enabled detection of a number of archaeal signatures in the human samples, suggesting archaea are essential constituents of the human microbiota [117], colonizing infants during the first year of life [118] and forming distinct communities across the human body [2,119].

The majority of the detected archaea in the human gastrointestinal tract are methane-producing organisms (methanogens), which possess the unique ability to respire H_2_ and produce methane as the main metabolic product under anaerobic conditions. In the human gut, the methanogens exist in a syntrophic relationship with bacterial species. Anaerobic bacterial fermentation results in the generation of short-chain fatty acids (SCFAs) such as acetate, propionate, and butyrate, as well as carbon dioxide and hydrogen gas [120]. Accumulation of H_2_ in the colon, however, inhibits bacterial energy production. By acting as a hydrogen sink and removing hydrogen gas, methanogens improve bacterial fermentation efficiency and allow more complete anaerobic degradation of organic material [120], thus confirming archaea as a keystone species essentially involved in metabolic processes and optimal energy yield of the entire human microbiota [121]. Methanogens in the human gastrointestinal tract account for up to 10% of all gut anaerobes, with *M. smithii* being the predominant archaeon, found in almost every subject [122], possibly due to its ability to establish syntrophic association with several bacterial species [123]. *M. stadtmanae* and *M. luminyensis* were detected in 30% and 4% of subjects tested, respectively [115,122]. Along with two candidate species “*Candidatus Methanomassiliicoccus intestinalis*” and “*Candidatus Methanomethylophilus alvus*”, several unknown members of the orders *Methanosarcinales*, *Methanobacteriales*, *Methanococcales*, *Methanomicrobiales*, and *Methanopyrales* were found to populate the human gut [124]. Besides these methanogenic archaea, members of the orders *Desulfurococcales*, *Sulfolobales*, *Thermoproteales*, *Nitrososphaerales*, and *Halobacteriales* have also been detected in the human intestine [124]. Recent publication utilizing archaea-specific methodology suggested an increased prevalence of *M. stadtmanae* in the majority of human samples, but failed to detect any nonmethanogenic archaeal lineages [112]. The only nonmethanogenic strains that were successfully isolated and characterized from the human gut are *Haloferax massiliensis* and *Haloferax assiliense*, demonstrating halophilic archaea can inhabit the human gut [125,126].

The potential role of archaea in human health and disease is a controversial subject. High prevalence of *M. smithii* in human population, as well as its low immunogenic potential, suggests this species is a typical commensal gut microbe [127,128]. The same finding was reported for *M. luminyensis* [129]. In fact, *M. luminyensis* was shown to be able to degrade trimethylamine (TMA), a compound associated with metabolic disorders such as trimethylaminuria [130], as well as reducing trimethylamine-N-oxide (TMAO) plasma levels, preventing the development of cardiovascular and chronic kidney diseases [131,132]. Thus, a potential use of *M. luminyensis* as an archaeal probiotic, or “archaebiotic”, was proposed to promote a positive effect of archaea on human health [133].

On the other hand, several studies suggest the role of archaea in the development of intestinal diseases. Methane, the end-product of methanogenesis, has been implicated in slowing down the intestinal transit resulting in constipation and gastrointestinal disorders [134,135]. In a population with irritable bowel syndrome (IBS), the subjects with the constipation-dominant disease (IBS-C) were shown to have a higher proportion of methane producers than individuals with the diarrhea-dominant disease (IBS-D) [135]. It was also shown that IBS-C involves an increase in *M. smithii* abundance [136] and a specific formulation of lovastatin, a fungal metabolite found to inhibit methane production in methanogenic microorganisms, was recently tested as a possible way to treat those suffering from IBS-C [137]. The methanogenic archaea are also associated with the etiology of IBD. A three-fold increase in the abundance of *M. stadtmanae*, which was shown to possess high immunogenic potential [127], was reported in IBD patients when compared to healthy individuals, suggesting this archaeon might be involved in pathologic conditions within the human gut [138]. Studies also indicated a lower proportion of patients were positive for methanogens in their gut compared to controls, primarily due to a reduction in the number of *M. smithii* in IBD subjects [138,139]. A recent report found significantly lower *M. smithii* levels among IBD patients compared to healthy individuals, with the levels returning to normal values in disease remission [140]. To conclude, the shift in archaeal populations associated with IBD could be related to bacterial dysbiosis and alteration in the intestinal nutritional environment (i.e., reduction of hydrogen gas and increase of short-chain alcohols such as methanol), favoring methylotrophic archaeal species, particularly *M. stadtmanae*, thus increasing the inflammatory response within the human gut [141].

The recent hypothesis on the role of archaea in human intestinal diseases suggests the butyric acid, a short-chain fatty acid (SCFA), as the essential component in the regulation of syntrophic archaea/bacteria biofilms in the gut. The archaeal overgrowth and increased removal of SCFA from the biofilms results in dysbiosis, triggering bacteria to become endoparasitic and enter intestinal epithelial tissues, which in turn leads to inflammatory processes in the human gut [142]. The “syntrophic imbalance hypothesis” is supported by the fact that methanoarchaea overgrowth is implicated in human diseases, including the IBS [143] and the levels of butyric acid are shown to decline in patients with IBS and IBD [144,145]. Moreover, one of the suggested treatments for intestinal disorders proposes utilizing butyrate-producing bacteria supplements to enhance intestinal epithelial barrier integrity [146]. In addition, the ryzophagy cycle in plant/symbiotic bacteria systems identified butyric acid as a signal molecule for maintenance of bacteria in biofilms, with the low concentrations of butyric acid inducing bacterial endoparasitism [147].

Although the research on the commensal viral and fungal constituents of human microbiota gained momentum recently, most microbiota studies still fail to include the archaea. The studies on human archaeome usually employ either cultivation or qPCR methodology [118,119,148], while 16S rRNA gene-based research often uses bacterial-targeted protocols and universal primer pairs to cover the broadest prokaryotic diversity [119,149]. The low abundance of archaeal DNA in human samples, inefficient cell lysis and DNA extraction, failure of the universal primers to fully detect archaeal signatures, as well as incomplete 16S rRNA gene databases, all represent methodological pitfalls of human archaeome analysis [112,150,151]. As archaea feature fundamentally different biology compared to their bacterial and eukaryotic counterparts, a specific methodology is required to investigate the human archaeome. Pausan et al. recently proposed an optimized detection method using specific 16S rRNA gene targeting primer pair combinations for NGS amplicon sequencing, as well as optimized qPCR protocols for quantifying the archaeal 16S rRNA gene to evaluate the bacteria/archaea ratios [152]. The proposed approach might prove useful as a standard operating procedure for detecting the full spectrum of archaeal diversity in human samples.

The archaea are a keystone species in the human microbiota with a profound impact on all aspects of human life. Although our current knowledge on the archaea is still mostly based on methodological concepts highly biased toward bacterial commensals, improved detection methodologies should enable future studies to further identify new archaeal taxa, assess their impact on the composition and function of the entire gut microbial community, as well as their contribution to human health or disease.

## 5. Eukaryotic Parasites

The pathogenic potential of commonly encountered intestinal parasites is well established. Mounting research evidence, however, suggests that instead of causing disease, a significant proportion of these organisms can be linked to maintaining intestinal homeostasis [4]. Eukaryotic parasites evolved to cause minimal harm to their human hosts [153], and some protozoan species can be considered a part of the human microbiota. Hamad et al. recently employed classical and molecular parasitological diagnostic methods to confirm the presence of over 15 different genera of protozoa (amoebozoans, flagellates, ciliates, stramenopiles, and apicomplexans) known to parasitize, but also commensalize the human gastrointestinal tract [154].

The role of protozoan parasites in the development and progression of IBD has been studied by several research groups, with the emphasis on *Blastocystis hominis* [155,156]. *B. hominis* is a common protozoan parasite in the gut found in both humans and animals, transmitted by the fecal–oral route and associated with various gastrointestinal disorders. Initial studies reported a significantly increased frequency of protozoa infection, with a high rate of *B. hominis*, in UC patients with persistent and intermittent disease activity as compared to the remission states [157]. Similarly, another study demonstrated *B. hominis* infection was associated with more severe symptoms and decreased disease treatment efficiency in patients with refractory ulcerative colitis [158]. A more recent study revealed specific isolates of *Blastocystis* that may exert pathogenic effects by disrupting gut microbiota [159]. This finding was in line with epidemiological data that linked virulent subtypes of *B. hominis* with intestinal dysbiosis [160]. However, contrasting results on the role of *B. hominis* in IBD were also reported. Studies revealed a lower burden of *B. hominis* (and *Dientamoeba fragilis*) in patients with UC when compared to the healthy individuals [161,162], while another report demonstrated a lower prevalence of *B. hominis* in patients with active UC compared to those in remission [163]. Additionally, *Blastocystis*-positive patients exhibited a higher abundance of *Clostridia* class, *Ruminococcaceae*, and *Prevotellaceae* families, as well as *Faecalibacterium* and *Roseburia* butyrate-producing bacterial genera, while *Enterobacteriaceae* were enriched in *Blastocystis*-free patients, suggesting *B. hominis* colonization was not associated with the colitis-specific dysbiosis but with an increased diversity of intestinal bacterial microbiota [164]. A recent study found no difference in *B. hominis* prevalence between the active and remission phases of the disease, suggesting that *B. hominis* does not play a role in UC flare-up [165]. Moreover, this study reported a higher abundance of *B. hominis* in the healthy group than in patients with UC, as well as milder disease symptoms for majority of *Blastocystis*-positive UC patients. Similar findings were published for IBS as well [166,167], thus implying *B. hominis* is a common constituent of healthy human microbiota exerting a protective role in gastrointestinal diseases [156]. Due to the proposed mutualistic interaction of *Blastocystis* and its host, some authors even suggested using the protist in a manner similar to that described for some intestinal helminths [168], in order to elicit a beneficial immunomodulatory response in patients with IBD [169]. Additional confirmation that *B. hominis* could be a commensal microorganism comes from a study in which patients with recurrent *Clostridium difficile* infections (rCDI) were treated using fecal microbiota transplantation (FMT) [170]. Although *Blastocystis*-positive fecal samples were previously excluded from FMT by many stool banks, resulting in a high rate (30%–50%) of donor exclusion, this study reported no adverse gastrointestinal symptoms nor any significant effect on the treatment outcome when *Blastocystis*-positive stool was used for the procedure.

Many protozoan parasites, however, are pathogenic species and can cause intestinal inflammation and disease. Colitis induced by *Entamoeba histolytica* may resemble CD [171,172]. Other parasites can complicate the course of IBD, i.e., cryptosporidiosis leads to increased hospitalization in children with either UC or CD [173]. Considering the latter, an association between *Cryptosporidium parvum* infection and gut dysbiosis (most notably perturbations of *Bacteroidetes*/*Firmicutes* ratio linked to chronic inflammatory processes) has been demonstrated in animal models [174]. On the other hand, the presence of the *Entamoeba* spp. (excluding the pathogenic *E. histolytica*) has been linked to increased bacterial diversity in the gut, as well as with the changes in the composition of the gut microbiome—more specifically, *Entamoeba* colonization was negatively correlated with inflammatory diseases and autoimmune disorders [175].

Finally, one of the major issues that hampers further exploration of the relationship between protozoan parasites and other microbiota is the deficiency of genomic data on intestinal protozoa and other parasites [4]. While genomic data is available in public databases for *Blastocystis* and *Dientamoeba*, there is scant ribosomal DNA sequences for some other gut parasites, even the common ones such as *Entamoeba coli* [4,176]. In addition, there is extensive genetic diversity in some common gut parasites, thus the associations between dysbiosis and various subtypes or genotypes are still not elucidated.

## 6. Conclusions

The significant role of non-bacterial microbiota in maintaining human homeostasis, as well as in disease etiology, is slowly unveiling (Table 1). The impact of these diverse fungal, viral, archaeal, and protozoan communities on human health needs to be determined in more detail in order to expand the current “bacteriocentric” view of human microbiota and provide more holistic understanding of the human superorganism.

To achieve this task, two important prerequisites are essential: (1) expanding fungal, viral, archaeal, and protozoan reference genomes in the currently available databases for reliable identification of those microorganisms; (2) establishing uniform methods of detection for each non-bacterial commensal population to ensure consistent and comparable evaluation of microbial abundance in different human body sites. The improved tools and the newly generated data would provide deeper insight on commensal non-bacterial communities and the possibilities of their exploitation in promoting human health and ameliorating disease. Although microbiome-directed therapy is still in its infancy, studies conducted thus far suggest that direct or indirect alterations in human virome and mycobiome, as well as changes in archaea and eukaryotic parasites may improve health outcomes in inflammatory diseases such as IBD.

However, the association of commensal fungi, viruses, archaea, and eukaryotic protozoa with the host in healthy/diseased states, as well as their interactions with each other, reflects a rather intricate nexus that transcends a mere “cause and effect” relationship. Experimental approaches should, therefore, be tailored to pinpoint where and how exactly these constituents of human microbiota play a role (i.e., whether at the disease onset, during early stages, or during active or latent disease) and account for a plethora of confounding factors which are currently pervasive in most of the studies. Although future research venues are paved with colossal challenges, the investments may pay off, as future applications of microbiome-based diagnosis, prognosis, treatment, monitoring, treatment, and prevention of the disease holds promise for a paradigm shift in translational and clinical medicine.

## Figures and Tables

**Table 1:** Major contributors of non-bacterial microbiota changes in IBD.

		IBD Type	Change	Reference
**Mycobiome**		CD + UC	↑ *Basidiomycota*/*Ascomycota* ratio	[53]
		CD + UC	↑ *Candida albicans*	[53]
		CD	↑ *Candida tropicalis*	[54]
		CD	↑ *Candida glabrata*	[55]
		CD	↑ *Gibberella moniliformis*	[55]
		CD	↑ *Alternaria brassicola*	[55]
		CD	↑ *Aspregillus clavatus*	[55]
		CD	↑ *Cystofilobasidiaceae* family	[55]
		CD + UC	↓ *Saccharomyces cerevisiae*	[53]
		CD + UC	↓ *Malassezia sympodialis*	[53]
		UC	↓ Fungal diversity	[53]
		CD + UC	↑ Fungal burden	[55,56]
		UC	↑ Fungal–bacteria interactions	[53]
		CD	↓ Fungal–bacteria interactions	[53]
**Virome**	**Phageome**	CD	↑ Phages infecting bacterial orders *Alteronomoadales* and *Clostridiales*	[87]
		CD	↓ *Microviridae* family	[89]
		CD + UC	↑ *Caudovirales* order	[88,90]
		CD + UC	↓ Phage diversity	[88,90]
	**Eukaryotic virome**	CD	↑ *Retroviridae* family	[87]
		UC	↑ *Pneumoviridae* family	[90]
		UC	↓ *Anelloviridae* family	[90]
		CD + UC	↑ *Herpesviridae* family	[97,98]
		CD + UC	↑ *Hepadnaviridae* family	[99]
		CD + UC	↑ *Hepeviridae* family	[99]
		UC	↓ *Polydnaviridae* family	[99]
		UC	↓ *Tymoviridae* family	[99]
		CD	↓ *Virgaviridae* family	[99]
**Archaeome**		CD + UC	↓ *Methanobrevibacter smithii*	[138,140]
		CD + UC	↑ *Methanosphaera stadtmanae*	[138]
**Eukaryotic parasites**		UC	↑ *Blastocystis hominis*	[157,158]
		UC	↓ *Blastocystis hominis*	[161,162,163,164,165]

## Abbreviations

IBD	inflammatory bowel disease
CD	Crohn’s disease
UC	ulcerative colitis
IBS	irritable bowel syndrome
SCFA	short-chain fatty acid
NGS	next-generation sequencing
FMT	fecal microbiota transplantation
rCDI	recurrent *Clostridium difficile* infection
